# A cross-sectional study of substance use in patients with schizophrenia hospitalized for relapse at the Ar-Razi Psychiatric Hospital in Salé, CHU Ibn Sina Rabat, Morocco

**DOI:** 10.11604/pamj.2022.41.107.30606

**Published:** 2022-02-08

**Authors:** Aboubacar Abderemane, Taher Moussa Ahmadou, Abderrazzak Khadmaoui, Siham Belbachir, Khalid Barkat, Ahmed Omar Touhami Ahami

**Affiliations:** 1 Biology and Health Laboratory, Cognitive-Behavioural Neuroscience and Nutritional Health Unit, Faculty of Science, Ibn Tofail University, Kenitra, Morocco,; 2 Laboratory of Genetics and Biometry, Faculty of Science, Ibn Tofail University, Kenitra, Morocco,; 3 Faculty of Medicine and Pharmacy, Mohammed V University, Rabat, Morocco; 4 Nutrition Dietetics, Ibn Sina University Hospital Centre (CHUIS), Ministry of Health, Rabat, Morocco,

**Keywords:** Schizophrenia, tobacco, cannabis, alcohol, addiction, Morocco

## Abstract

**Introduction:**

substance use disorders are becoming increasingly common among schizophrenic patients and often raise problems in their care. This work aims to assess the rate and level of substance use in schizophrenic patients hospitalized for relapse and to identify factors associated with this comorbidity.

**Methods:**

we conducted a cross-sectional study of 115 patients with schizophrenia selected by convenience sampling in the Psychiatric Hospital of Salé. A questionnaire was used to collect socio-demographic data, evolutionary and prognostic criteria of the disease. Tobacco use was assessed with the fagerström test for nicotine dependence (FTND), cannabis use was assessed with the cannabis abuse screening test (CAST) and alcohol use was assessed with the alcohol use disorders test (AUDIT).

**Results:**

the results showed that tobacco was the most consumed substance, followed by cannabis and alcohol. Certain factors such as gender (P<0.016) and family smoking history (P<0.045) were significantly associated with the scale (FTND). Other factors such as social life (P<0.05) and the educational level (P<0.004) showed a significant association with CAST. Only a history of suicide attempts was significantly associated with the scale (AUDIT).

**Conclusion:**

the results confirm that the majority of schizophrenic subjects are psychoactive substance users and that a good number of them are dependent on substances. Early outpatient follow-up in specialized drug treatment centers could improve the health status of these patients.

## Introduction

Schizophrenia is a disease that occurs almost everywhere in the world [1]. Over a lifetime, the probability of developing schizophrenia is approximately 0.6 to 1% and the average annual incidence is about 15 new illnesses per 100,000 people [1]. In Morocco, more than 200,000 people aged 15 and above, suffer from schizophrenia, and psychiatrists are often faced with the problem of co-morbidity between schizophrenia and addictive behaviors [2]. The majority of epidemiological studies validate the hypothesis that addictive comorbidities in schizophrenia affect the majority of patients [3]. According to epidemiologic catchment area (ECA) data, the incidence of drug addiction on schizophrenia is 47% in a lifetime, including 37% with alcohol abuse and dependence disorder and 27.5% for other drugs [4]. The use of psychoactive substances is not without consequences for the emergence of schizophrenia, and cannabis remains the most incriminated substance so far. A Swedish study has shown that cannabis consumers were 2.4 times more likely to develop schizophrenia than non-users [5]. In 62.1% of cases, excessive cannabis use preceded or was accompanied by the onset of schizophrenia, and in 34.6% of cases, the onset of both disorders occurred simultaneously in the same month [6]. Nevertheless, cannabis use is not sufficient to cause schizophrenic disorders. The latter would come from the complexity of both genetic risk factors and other environmental risk factors [7]. However, the association of schizophrenia with the use of psychoactive substances negatively influences the quality of life of patients as well as their life expectancy [3]. Patients with schizophrenia have a higher mortality rate than the general population, for natural causes (cancers and cardiovascular diseases) and suicides, in the forefront, according to recently published European studies [8]. On the contrary, other studies show that smoking does not only have negative effects on schizophrenics, but it also alleviates negative symptoms and reduces their cognitive symptoms [9,10]. The purpose of this study was to assess the rate, as well as the level, of psychoactive substance use in patients with schizophrenia hospitalized for relapse and identify factors associated with this comorbidity.

## Methods

**Study design and setting:** we conducted a descriptive cross-sectional study of patients with schizophrenia, whether or not they used psychoactive substances, over a period of six months, more precisely between September 2019 and March 2020, at the Ar-Razi Psychiatric Hospital of the Center of Ibn Sina University Hospital of Rabat (CHUIS), Morocco.

**Study population:** it involved all patients, men, and women, diagnosed with schizophrenia by their psychiatrist. Patients who were non-consenting, uncooperative, severely disorganized or deficient, or had other mental illnesses other than schizophrenia were excluded from the study. After informing them about the research protocol, informed consent was sought from them before including the patients in the study.

**Data collection:** a questionnaire was used to collect socio-demographic data, evolutionary and prognostic criteria of the disease (age, sex, duration of the disease, and suicidal history) as well as characteristics related to psychoactive substance use. Tobacco use was assessed through the Fagerström test for nicotine dependence (FTND) scale developed by Fagerström in 1978 as the Fagerström tolerance questionnaire (FTQ) is designed to measure physical dependence on nicotine [11]. A revised version of FTQ was proposed by Heatherton *et al*. in 1991 and remains the most widely used scale in the world for the assessment of physical nicotine dependence [12]. It consists of only six questions, instead of eight with a total score ranging from 0 to 10 [13]: i) a score of (0-2) is equivalent to very low dependency; ii) a score of (3-4) is equivalent to low dependency; iii) a score of 5 is equivalent to a medium dependency; iv) a score of (6-7) is equivalent to a high dependency; v) a score of (8-10) is equivalent to a very high dependency.

However, when entering the data, we classified the scores into two categories. A score of less than 5 was defined as low dependency and a score of 5 or more was defined as medium or high dependency [14]. Cannabis use was assessed using the cannabis abuse screening test (CAST), developed by the French observatory for drugs and drug addiction (OFDT) in 2002, which is one of the most frequently used scales for the identification of problematic cannabis use [15]. The CAST is a 6-item scale in which a score of less than 3 defines users with no risk of dependence, a score of 3 to 6 defines users with a low risk of dependence and finally a score of 7 or more defines users with a high risk of dependence [16]. We also classified the scores into two categories where a score greater than or equal to 7 was defined as a high risk of dependence and a score less than 6 was defined as low risk or no dependence on cannabis. Alcohol use was assessed by using the alcohol use disorders test (AUDIT). This is a 10-item scale developed by the WHO in which the answers to each question are rated on a scale ranging from 0 to 4, giving a maximum score of 40 [17]. A score of 5 or less indicates risky drinking, a score between 6 and 8 indicates harmful drinking for men (7 instead of 8 for women). Lastly, a score between 9 and 12 or more indicates probable alcohol dependence in men (11 instead of 12 in women) [17]. However, we again classified the scores into two categories, where a score of less than or equal to 8 (7 for women) was defined as harmful or risky drinking and a score greater than or equal to 12 (11 for women) was defined as probable alcohol dependence. The use of psychotropic drugs (benzodiazepines) was assessed using the cognitive scale for benzodiazepine attachment (ECAB questionnaire). The ECAB is a 10-item questionnaire graded 1 or 0 to assess benzodiazepine dependence. A score of 6 or higher indicates dependence with an estimated sensitivity of 94% and specificity of 81% [18].

**Sampling procedure and sample size:** the participants of the study were selected based on a non-probability convenience sampling method. However, as we do not know the exact prevalence of schizophrenia in the study area, the sample size was defined by the number of cases admitted to the ward during the six months of the study.

**Sample size and statistical analyses:** the study involved a sample of 115 patients hospitalized for schizophrenic relapse. The data collected were entered in Excel, after filtering and coding were re-entered on the support of statistical analysis SPSS version 23. Data were submitted to descriptive analyses (mean, the standard deviation for quantitative variables, and percentage for qualitative variables) and conjoint analyses (classical parametric tests, more precisely the chi-square test). Variables with a p value <0.05 were considered statistically significant.

**Ethical approval:** the approval to conduct this study was granted by the management of the Ibn Sina Hospital CHUIS in Rabat. Indeed, a written agreement was received from the management of the center of Ibn Sina University Hospital of Rabat (CHUIS)- Rabat for the conduct of the study. Thus, the informed consent of patients was also obtained before the inclusion of patients in the study.

## Results

**Socio-demographic characteristics:** the study included 115 schizophrenic patients of whom 77.4% (n=89) were male, the sex ratio was not balanced (3.42), it was in favor of males. The mean age of patients was 30.9 ± 0.83 years (minimum age =16 years; maximum age =62 years), of whom 82.6% (n=95) were single. Nevertheless, 61.7% of these patients had reached high school education and 38.3% a higher level. However, 67.5% of these patients didn't have any professional activity, while more than 79% came from the urban environment. Nonetheless, 39.1% of patients had a family history of psychiatry and 3.5% had a family history of suicide attempts. However, 18.3% of these patients had a history of suicide attempts. On the other hand, 55.8% of patients had been hospitalized more than once with an average length of hospitalization of 38 days.

**Figure 1:**
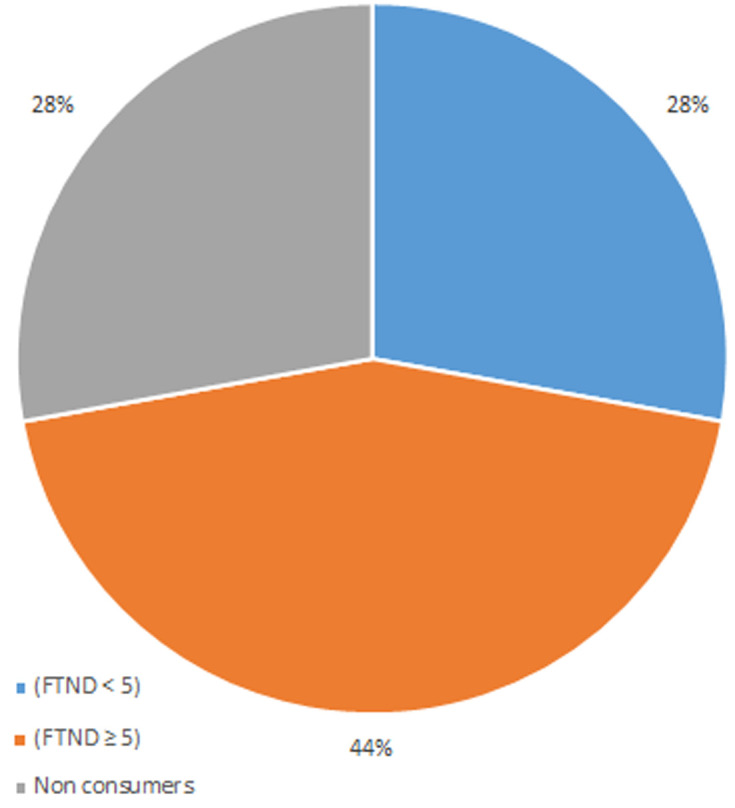
distribution of tobacco use and level of consumption according to the Fagerström scale

**Characteristics of tobacco use:** Figure 1 summarises the distribution of tobacco consumption and the level of consumption according to the Fagerström scale. According to the scores obtained by the Fagerström scale, the frequency of tobacco consumption among surveyed patients was 72%, distributed as follows: 44% of our sample manifest a medium or strong dependence on tobacco (FTND≥ 5), 28% a weak dependence (FTND <5) and 28% were non-smoker. Indeed, 12% of smokers declared to consume only tobacco, the rest of the smokers consumed tobacco and other substances (cannabis, alcohol e.t.c). Nonetheless, most of the patients started smoking at a young age (mean 16.6±0.44 years). Indeed, the majority of our patients started smoking before the occurrence of the disease, i.e. 83.3% of smokers.

**Relationship between schizophrenia and smoking:** Table 1 presents the results of the chi-square test for variables showing a significant correlation. On the contrary, variables such as gender, family history of smoking, and reason for smoking were found to be highly associated with the Fagerström test, with p values of (p<0.016); (p<0.045) and (p<0.000) respectively. Indeed, for the gender trait, 41.09% of male smokers were found to be dependent (score ≥ 5) as compared to 20% of female smokers. However, among those who responded that they had a history of smoking in the family (n=65), 33.85% had a moderate or strong dependence compared to 55.55% who said “no”. In addition, 45.45% of dependent respondents answered that it was curiosity that pushed them to use tobacco, 28.26% answered that it was their environment.

**Table 1:** chi-square test of independence between the Fagerström test and certain factors such as gender, history, and reason for use

		Fagerström		Total	Chi-square (p-value)
		(score≥5)	(score<5)		
Gender	Male	41.09% (n=30)	58.90%(n=43)	73	8.25 (p <0,016)
	Female	20% (n=2)	80%(n=8)	10	
History of tobacco use in the family	Yes	33.84% (n=22)	66.15%(n=43)	65	6.18 (p<0,045)
	No	55.55% (n=10)	44.44%(n=8)	18	
Reason for tobacco use	Curiosity	45.45% (n=10)	54.45%(n=12)	22	93.22 (p<0,000)
	Boredom	80% (n=4)	20%(n=1)	5	
	Environment	28.26% (n=13)	71.73%(n=33)	46	
	Other reasons	50% (n=5)	50%(n=5)	10	
Total		32	51	83	

**Figure 2:**
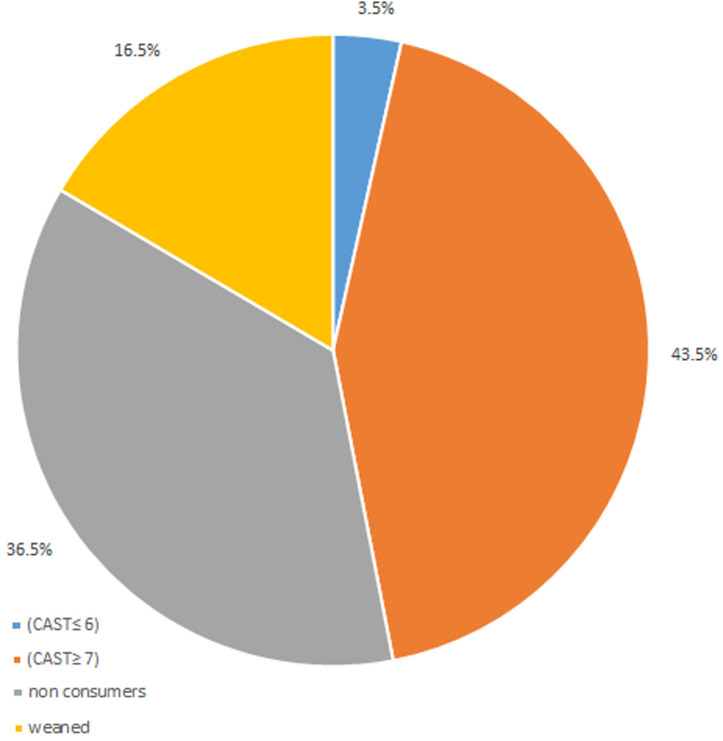
distribution of cannabis use and level of consumption according to the CAST questionnaire

**Characteristics of cannabis use:** Figure 2 summarises the distribution of cannabis use and level of use according to the CAST questionnaire. It shows that among cannabis users, the majority of our patients, 43.5%, had a high risk of cannabis dependence (CAST ≥ 7), 3.5% a low risk of dependence (CAST ≤ 6) and 16.5% were former smokers. Furthermore, most cannabis smokers started using it before the onset of the disease, 72.6% of cannabis users. On average, the age at onset of cannabis use was 17.5 ± 0.47 years.

**Relationship between schizophrenia and cannabis use:** Table 2 shows the results of the chi-square test of independence between the CAST test and certain variables. Indeed, only educational level and social life were significantly associated with CAST, with p-values of 0.004 and 0.05 respectively. Nevertheless, 94.11% (n=48) of patients among those living with their family showed a high risk of cannabis dependence according to the CAST scores. However, no association was observed between the gender variable and the latter (p<0.56).

**Table 2:** chi-square test of independence between the cast questionnaire and certain factors such as gender, social life, and the level of education

		CAST		Total	Chi-square (p-value)
		CAST ≤6	CAST ≥7		
Gender	Male	8%(n=4)	92%(n=46)	50	0.35 (p<0.56)
	Female	0%(n=0)	100%(n=4)	4	
Social living	Alone	33.33%(n=1)	66.66%(n=2)	3	3.11 (p<0.05)
	With the family	6%(n=3)	94.11%(n=48)	51	
Educational level	Never in school	100%(n=1)	0%(n=0)	1	13.11 (p<0.004)
	Primary	0%(n=0)	100% (n=5)	5	
	Secondary	7.40% (n=2)	92.59%(n=25)	27	
	University	4.76%(n=1)	95.23%(n=20)	21	
Total		4	50	54	

**Figure 3:**
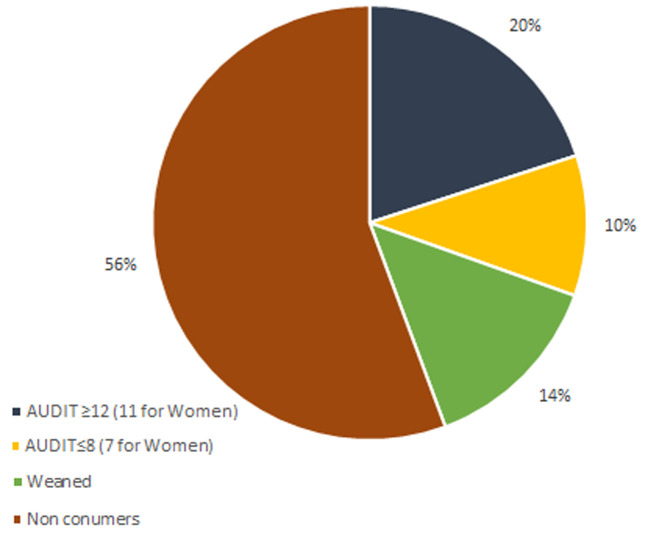
distribution of alcohol consumption and level of consumption according to the AUDIT scale

**Characteristics of alcohol consumption:** Figure 3 shows the distribution of alcohol consumption and the level of consumption according to the AUDIT scale. It also shows that 44% of them had already consumed alcohol, of which 14% were former drinkers and 20% were alcohol dependent, according to the scores obtained by the AUDIT questionnaire (Figure 3). However, the average age of alcohol initiation was 18.04±0.0.52 years. Moreover, the majority of patients started drinking before the onset of the disease (62.74%).

**Relationship between schizophrenia and alcohol consumption:** Table 3 shows the results of the chi-square test for the independence of the AUDIT scale and certain variables. Indeed, variables such as gender and environment didn't show a significant relationship, and the variable antecedent suicide attempt was at the limit of significativity (p<0.05). Furthermore, 9 out of 10 patients with probable alcohol dependence according to the AUDIT questionnaire already had a history of suicide attempts, and 63.33% of alcohol-dependent patients lived in urban areas. Nonetheless, no association was found between the variables gender, environment, and the AUDIT questionnaire (p<0.52). Apart from tobacco, cannabis, and alcohol, we were able to identify other substances used by patients, including psychotropic drugs, lysergic acid LSD (diethyllysergamide or LSD), cocaine, ecstasy, organic solvent, and maajoune (made from hemp resin and aphrodisiac substances). Hence 14% of patients were users of psychotropic drugs such as benzodiazepines and only one patient was dependent on benzodiazepines as measured by the ECAB scale. For LSD, only one patient was a consumer. The distribution according to the use of other substances was estimated as follows: 13% for cocaine, 10.4% for ecstasy, 9.6% for organic solvents, and 4.3% for maajoune. We were unable to make a correlation analysis with the other characteristics given the reduced number of users of these substances.

**Table 3:** chi-square test of independence between the audit questionnaire and certain factors such as gender, environment, and history of suicide attempts

		Audit		Total	Chi-square (p-value)
		Audit≥ 12 (11 for woman)	Audit≤ 8 ( 7 for woman)		
Gender	Male	65.51%(n=19)	34.48%(n=10)	**29**	**0.4 (p<0.52)**
	Female	80%(n=4)	20%(n=1)	**5**	
Area	Urban	63.33%(n=19)	36.66%(n=11)	30	2.16 (p<0.06)
	Rural	100%(n=4)	0%(n=0)	4	
Attempted suicide	Yes	90%(n=9)	10%(n=1)	10	3.23 (p<0.05)
	No	58.33%(n=14)	41.66%(n=10)	24	
Total		23	11	34	

## Discussion

The results of this study show that more than half of schizophrenic patients are psychoactive substance users and that tobacco remains the most commonly used substance, followed by cannabis and then alcohol. Moreover, the majority of patients who use psychoactive substances began their consumption before the onset of the illness, i.e. 83.3% of smokers, 72.6% of cannabis smokers, and 62.7% of alcohol users, which raises the question of the role of these substances in the emergence of schizophrenia. Several studies point out that the use of psychoactive substances precedes the onset of the disease [19,20]. Of the 115 schizophrenic patients in our study, 77.4% were male and 22.6% were female. The female gender was under-represented in our sample, while the male gender was in the majority. In general, therefore, men were predominant [21]. The majority of patients (82.6%) were single, which is similar to what is found in the literature [19]. This high frequency among single people can result in consequences on patients´ social life, preventing them from starting a relationship with the opposite sex [22]. Concerning the educational and professional level, 61.7% of patients had secondary level education, 38.3% had a higher level and 67.5% didn´t have any professional activity, which can be explained by the importance of cognitive abilities and their primordial role in social and professional insertion [23]. Although the new antipsychotics seem to show a lot of improvement on cognitive faculties compared to the former treatments, pharmacological treatments can lead to think that they influence the intellectual functioning and the academic life of patients [24]. Similarly, 79% of patients in our study were from urban areas compared to 21% from rural areas. We found a higher incidence rate in urban areas than in rural areas [25].

Regarding the family psychiatric history of our sample, 39.1% confirmed having a family member with a psychiatric history. Moreover, a child is four times more likely to develop bipolar disorder if one parent has bipolar disorder. For depressive disorders, the average heritability was 15-25%, and if one sibling had schizophrenia, the other had a 6% chance of also being affected [26]. Among the most devastating consequences of schizophrenia, suicide attempts among schizophrenic patients remain at the forefront. Our study shows that 18.3% of schizophrenic patients reported at least one suicide attempt compared to 20-50% in the literature [27]. The results of our study show that tobacco is the most consumed substance, i.e. 73% of our sample, of whom 64.28% showed a medium or strong dependence according to the scores obtained by the Fagerström scale and in 12% of cases, tobacco is consumed alone. The study by Delignère A-L *et al*. 2018 showed that 64.1% of schizophrenics used tobacco and 60.3% of smokers had a moderate or strong dependence according to the Fagerström scale [14]. Elghazouani F *et al*. in Fez, Morocco in 2015 showed that 72.2% smoked cigarettes and 13% of cases, tobacco was consumed alone [28]. However, Dervaux and Laqueille in 2005 showed that the frequency of smoking in schizophrenic patients is between 60 and 90% compared to 23 to 30% in the general population [29]. Similar to the study by Ferchiou A *et al*. in 2010 which showed 20 to 30% of tobacco users in the general population versus 70% in schizophrenic patients [30]. A meta-analysis estimated the prevalence of tobacco use to be 62% in schizophrenic patients, which is much higher than in the general population [31].

Indeed, the majority of patients started smoking before the onset of the disease (83.3% of our population). In addition, many studies show that the use of psychoactive substances precedes the disease in most cases [19,28,32]. On the other hand, gender was found to be strongly associated with tobacco dependence: 41.09% of male smokers were found to be dependent compared to 20% of female smokers (p<0.016). The same trend was found in several studies [14,33,34]. Family smoking history and reason for smoking were also found to be strongly associated with the Fagerström test in our study. The study by Lyons *et al*. showed that smoking was not only caused by the disease but also by the existence of a family vulnerability to nicotine [35]. Bersani *et al*. showed that the prevalence of cannabis use, which is currently the most widely used illicit drug in the world, is 4.6 times higher in schizophrenic patients than in the general population [36,37]. The analysis of our results shows that the frequency of cannabis use is 63.5%, and the majority of patients are addicted according to CAST. Moreover, cannabis users started using it before the onset of the disease. The first psychotic episodes appeared on average at least 5 years after the first use of cannabis [38]. People who started using cannabis regularly at a very young age are twice as likely to develop a psychotic disorder such as schizophrenia in the future [39]. Only the level of education (p<0.004) and social life (P<0.05) were significantly associated with cannabis abuse screening test (CAST). The majority of patients with a high level of cannabis dependence according to CAST had a secondary or university education and mostly lived with their families. However, other studies showed that patients with schizophrenia and cannabis use disorder had a lower level of education compared to non-users [40,41]. Indeed, the majority of cannabis users in our live sample lived with their families, and this form of social life was found to be strongly associated with CAST (p<0.05). The study by Tsuang *et al*. showed that cannabis abuse, especially marijuana, was influenced by some family environmental factors [42]. However, no significant relationship was found between these variables: family cannabis use history and gender with cannabis dependence according to the CAST.

Concerning alcohol consumption, the analysis of the results shows that the majority of current consumers were alcohol-dependent, as indicated by the AUDIT questionnaire. The 2012 study by Rane *et al*. in Goa, India, showed that the one-year prevalence of alcohol consumption was 16.8%, while hazardous drinking was 5.7% and alcohol dependence was 2.5% [43]. In our study, 68% of current consumers and 20% of the study population showed alcohol dependence, based on the AUDIT questionnaire. A meta-analysis of 60 studies published between 1996 and 2008 showed that the current and lifetime prevalence of alcohol use disorders was 9.4% and 20.6% respectively [44]. Others found that the prevalence of alcohol consumption was estimated at 47.2% of whom 25% were occasional users [28]. Delignère A-L *et al*. found that 21.7% of alcoholic schizophrenic patients suffered from problematic use of alcohol according to the AUDIT questionnaire and that the age of first alcohol use occurred on average at 17.7 years [14], which is similar to the results of our study which showed that the average age of onset of first alcohol consumption was 18.04 ± 0.52 years. We also noted the presence of a significant link between antecedent suicide attempt (variable) and probable alcohol dependence according to the AUDIT. The study by Hong-Chul Bae *et al*. in 2015 showed a significant association between AUDIT and suicide attempts and suicidal ideation [45] which is consistent with our study. Indeed, some studies found that alcohol abuse was associated with high rates of suicidal behavior [27,46-48]. Thomas P *et al*. in 2016 found that suicide was the leading cause of death and that substance and alcohol users were 2 to 3 times more likely to suffer unnatural mortality [3]. It is the only substance significantly associated with suicide attempts in patients with schizophrenia according to the study by McLean *et al*. [49].

**Limitations of the study:** as the study involved a non-probability convenience sample of small size, our results, do not represent and cannot be generalized to the national level. The absence of biological examination to confirm the use of psychoactive substances, as well as the small number of patients using psychotropic drugs, ecstasy, cocaine, organic solvents, and maajoune, constitute the limitations of this work.

## Conclusion

The combination of schizophrenia and psychoactive substance use is particularly frequent and is currently a real public health problem that is attracting the attention of health professionals. Cannabis is the most consumed illicit drug in our study, and this could be explained by its easy availability in the territory. The majority of patients using these substances started using them before disease onset and some socio-demographic factors are associated with substance dependence, namely environment, social life, family history of use e.t.c. This study highlights that the environment not only contributes to the emergence of schizophrenia in predisposed subjects, but can also play a role in the development of certain substance use disorders.

### What is known about this topic

- The frequency of psychoactive substance use is higher among people with schizophrenia than in the general population;
- This co-morbidity affects the quality of life of patients as well as their life expectancy;
- Patients with schizophrenia using psychoactive substances have many schizophrenic relapses, resulting in a high number of hospitalizations.

### What this study adds

- This study confirms that psychoactive substance use affects the majority of patients suffering from schizophrenia;
- Our results have identified a statistically significant association between psychoactive substances dependency and certain socio-demographic factors;
- The results of our study show the need to provide care for dependent patients in specialized addiction care centers in addition to their psychiatric care.
